# Differential Cellular Interactome in Schizophrenia and Bipolar Disorder—Discriminatory Biomarker Role

**DOI:** 10.3390/antiox12111948

**Published:** 2023-11-01

**Authors:** Iván Menéndez-Valle, Cristina Cachán-Vega, José Antonio Boga, Laura González-Blanco, Eduardo Antuña, Yaiza Potes, Beatriz Caballero, Ignacio Vega-Naredo, Pilar Saiz, Julio Bobes, Paz García-Portilla, Ana Coto-Montes

**Affiliations:** 1 Instituto de Investigación Sanitaria del Principado de Asturias (ISPA), Av. del Hospital Universitario, s/n, 33011 Oviedo, Asturias, Spain; 2 Instituto de Neurociencias (INEUROPA), University of Oviedo, Julián Clavería, s/n, 33006 Oviedo, Asturias, Spain; 3 Servicio de Inmunología, Hospital Universitario Central de Asturias (HUCA), Av. del Hospital Universitario, s/n, 33011 Oviedo, Asturias, Spain; 4 Department of Cell Biology and Morphology, Faculty of Medicine, University of Oviedo, Julián Clavería, s/n, 33006 Oviedo, Asturias, Spain; 5 Servicio de Microbiología, Hospital Universitario Central de Asturias (HUCA), Av. del Hospital Universitario, s/n, 33011 Oviedo, Asturias, Spain; 6 Servicio Regional de Investigación y Desarrollo Agroalimentario (SERIDA), Ctra. AS-267, 33300 Villaviciosa, Asturias, Spain; 7 Departament of Medicine, Faculty of Medicine, University of Oviedo, Julián Clavería, s/n, 33006 Oviedo, Asturias, Spain

**Keywords:** cellular death, mitochondria, oxidative stress, proteasome, psychiatric disorder, reticulum stress

## Abstract

Schizophrenia (SCH) and bipolar disorder (BD) are two of the most important psychiatric pathologies due to their high population incidence and disabling power, but they also present, mainly in their debut, high clinical similarities that make their discrimination difficult. In this work, the differential oxidative stress, present in both disorders, is shown as a concatenator of the systemic alterations—both plasma and erythrocyte, and even at the level of peripheral blood mononuclear cells (PBMC)—in which, for the first time, the different affectations that both disorders cause at the level of the cellular interactome were observed. A marked erythrocyte antioxidant imbalance only present in SCH generalizes to oxidative damage at the plasma level and shows a clear impact on cellular involvement. From the alteration of protein synthesis to the induction of death by apoptosis, including proteasomal damage, mitochondrial imbalance, and autophagic alteration, all the data show a greater cellular affectation in SCH than in BD, which could be linked to increased oxidative stress. Thus, patients with SCH in our study show increased endoplasmic reticulum (ER)stress that induces increased proteasomal activity and a multifactorial response to misfolded proteins (UPR), which, together with altered mitochondrial activity, generating free radicals and leading to insufficient energy production, is associated with defective autophagy and ultimately leads the cell to a high apoptotic predisposition. In BD, however, oxidative damage is much milder and without significant activation of survival mechanisms or inhibition of apoptosis. These clear differences identified at the molecular and cellular level between the two disorders, resulting from progressive afflictions in which oxidative stress can be both a cause and a consequence, significantly improve the understanding of both disorders to date and are essential for the development of targeted and preventive treatments.

## 1. Introduction

Schizophrenia (SCH) and bipolar disorder (BD), paradigms of severe mental disorders, are currently, because of their incidence and their high disabling potential, one of the greater challenges, if not the greatest, facing institutions and the international scientific community in the field of mental public health [1]. According to data from the World Health Organization (WHO), the lifetime incidence of these disorders combined is more than 1% of the world population, which represents more than 80 million people worldwide, with SCH and BD being the third and fifth cause of disability in young adults, respectively [2]. This, added to the large potential loss of life years related to these disorders [3], makes the economic cost for public health services very important and the social cost unbearable.

SCH and BD share important genetic risk loci, although a genetic basis for the observed differences between the two disorders has also been described [4]. In addition, environmental epigenetic modifications [5] have broad effects on the occurrence of these diseases [6]. This complexity together with the lack of knowledge of the molecular alterations present in both disorders mean that the pathophysiological mechanisms underlying both disorders are still unknown.

The concept of the interactome refers to the study of proteins that, within their subcellular environment, establish connections or protein complexes with other proteins, forming the basis of cellular processes and interactions between organelles. These subcellular protein networks can interact with each other, and, in turn, these interactions can be modulated or controlled by the expression of other proteins [7]. The different modulations that each pathology causes in these mechanisms offer an unparalleled scenario for detecting predictive markers [8].

Even taking into account the efficacy of the cellular interactome in biomarker determination, the collection of central nervous system (CNS) samples is very limited. Classically, the study of CNS-related diseases has been approached using postmortem brain samples, which are subject to various artifacts related to medication, cause of death, agonal state, and postmortem interval [9,10], with the added difficulty of subsequently collecting such samples in vivo. Currently, many studies focus on blood cells, in particular, the peripheral blood mononuclear cell (PBMC) phase, consisting mainly of lymphocytes and monocytes, which have the capacity to express almost all genetic material [11]. As they circulate throughout the body, they are able to respond to diffusible signals, such as hormones or nutrients, modulating their gene expression and acting as true sentinels capable of reflecting the gene expression profiles of various pathologies [11]. In addition, this condition allows for the simulated collection of patient plasma, a recognized biomarker donor that can also provide essential information in neurological diseases, whose information can, in turn, complement that obtained in PBMC [12].

Multiple mental illnesses have been described to be associated with increased oxidative stress [13,14], including SCH [15] and BD [16]. However, although the cellular damage caused by this oxidative imbalance [17] and its consequent disruption of cellular homeostasis [18] has been amply demonstrated in mental illnesses, no further study has been made of the trace that this increase in free radicals progressively leaves within the cell, limiting itself, in these mental illnesses, to confirming the appearance of inflammation as a necessary and well-known response to the prevailing oxidative stress [19]. But whether the cell responds to oxidative stress by increasing its own, where these free radicals come from, and what consequences they will have on its functioning (in short, what are the characteristic features of the interactome of both disorders) are completely unknown at present and which we will try to answer in this article.

Focusing on the cellular interactome, if the development of the disease is accompanied by an increase in oxidative stress, the role of mitochondria as either an initiator or mediator of free radical production is assured. Mitochondria are the main free radical-producing organelles in the cell, and their incoordination, malfunctioning, or cellular alteration has the immediate consequence of increasing oxidative stress [20] accompanied by a reduction in ATP synthesis, which decisively conditions cellular functioning [21]. In turn, cellular activity is based on two fundamental pillars, namely energy synthesis and protein production, and one depends on the other. Thus, energy imbalance leads to disruption of endoplasmic reticulum (ER) homeostasis and induction of ER stress. This imbalance in ER function results in increased production of misfolded proteins that require overexertion of the proteasome, the proteolytic complex responsible for their degradation. If the level of these defective proteins increases, alarms are triggered in the ER, activating the unfolded and misfolded protein response (UPR), which consists of three cascades of action initiated from the ER, inositol-requiring protein 1 (IRE1α) activation of transcription factor 6 alpha (ATF6α) and RNA-dependent protein kinase such as ER eukaryotic translation initiation factor 2 alpha kinase (PERK), which is also dependent on eukaryotic initiation factor 2 alpha (eIF2α) [22]. These three pathways favor a multidimensional response that triggers the activation of antioxidant, anti-inflammatory, and broadly transcriptional responses responsible for restoring protein synthesis capacity and cellular homeostasis. If necessary, important survival mechanisms such as autophagy can be triggered, and finally, when these recovery systems are not sufficiently effective, apoptosis is triggered to induce cell death [23].

This concatenated cellular response, which is not free of variability depending on the pathology [23,24,25], shows the real scenario that allows us not only to delve deeper into the mechanistic alterations, but also to detect potential differential markers between the two, by studying each of the possible mechanisms affected according to the key proteins that characterize them, such as LC3-II, Beclin-1, and p62 in the case of macroautophagy [26,27], or IRE-1α, PERK, and eIF2α for unfolded protein response [28]. On this basis, the cellular interactome alterations detected in patients with SCH and BD in cells at the systemic level have been the focus of the present article, in which clear differences between the two disorders have been demonstrated, supporting the possibility of molecular discrimination between them.

## 2. Materials and Methods

### 2.1. Subjects

#### 2.1.1. Experimental Design

This is a clinical staging for patients with SCH and BD. The study was carried out in 50 subjects (54% men, 46% women), divided into 3 groups: a control group (CON) of 14 individuals not previously diagnosed with any severe mental disorder (SMD) from the Centro Comunitario de Sangre y Tejidos de Asturias and 36 individuals with SMD, who were then divided into 22 diagnosed with SCH and 14 diagnosed with BD who are in treatment at the Mental Health Centers I and II of Health Area IV (MHCI and MHCII) (Oviedo, Asturias, Spain).

All patients were over 17 years of age, had a diagnosis of SCH or BD according to DSM-IV-R criteria, and were on stable maintenance treatment for at least 3 months prior to inclusion in the study. The diagnoses of SCH and BD were made by a psychiatrist and confirmed by the SCID clinical interview. Exclusion criteria for both groups were (1) somatic comorbidities, both acute (acute infection, fever, allergic or inflammatory processes) and chronic (cancer, autoimmune diseases, chronic infections), that could interfere with inflammatory parameters; (2) treatment with immunosuppressants or vaccines during the 6 months prior to inclusion; and (3) treatment with anti-inflammatory medications 2 days prior to blood collection. Exclusion criteria for control subjects also included a history of mental disorder. The study received the approval of the Clinical Research Ethics Committee of the Hospital Universitario Central de Asturias in Oviedo, Spain (Ref. 36/2012 and 25/2014). All participants gave their written informed consent before enrollment.

#### 2.1.2. Blood Collection

Blood samples were collected by venipuncture after an overnight fast, and hemogram analysis was performed at the MHCI and MHCII. Furthermore, collected blood samples were fractionated into plasma, erythrocytes, and PBMC in a Ficoll-Paque Plus from Cytiva (Marlborough, MA, USA) following the manufacturer’s instructions and stored at −80 °C until further analysis. The Bradford method [29] was used to measure protein concentrations.

#### 2.1.3. Clinical Parameters

To study and assess possible relationships between obtained results and clinical data, the following values were included in the study: body mass index (BMI), disability, antipsychotics, and total scores from the psychometric evaluation, including the Clinical Global Impression-Severity scale (CGGI-S), Positive and Negative Syndrome Scale (PANSS), Hamilton Depression Rating Scale (HDRS), Hamilton Anxiety Rating Scale (HARS), and Young’s Mania Rating Scale (YMRS). In the case of healthy controls, we were provided with a complete blood count. Some of these data are shown in Table 1.

### 2.2. Procedures

#### 2.2.1. Oxidative Stress Studies

Lipid peroxidation (LPO) was determined in plasma by measuring the reactive aldehyde malondialdehyde (MDA) and 4-hydroxy-2-(E)-nonenal (4-HNE) levels using the Lipid Hydroperoxide (LPO) assay kit from MilliporeSigma Calbiochem (Saint Louis, MO, USA) [30]. Total antioxidant activity (TAA) was determined using the 2,2′-azino-bis (3-ethylbenzothiazolin-6-sulfonic acid) radical cation method (ABTS) [31]. Superoxide dismutase (SOD) activity was measured in erythrocytes according to the method developed by Martin and colleagues [32]. Catalase (CAT) activity was assayed in erythrocytes using the method reported by Lubinsky and Bewley [33].

#### 2.2.2. Inflammation Studies

IL-6 (Human IL-6 ELISA Kit, Gen-Probe Diaclone SAS, Besancon, France) and TNF-α (TNF alpha Human ELISA Kit, Invitrogen Corp., Carlsbad, CA, USA) were measured in plasma using commercially available enzyme-linked immunosorbent assays following the manufacturer’s instructions.

#### 2.2.3. Western Blotting

Western blot immunoassays of the PBMC were performed according to the instructions previously described by our research group [24] using the respective primary and secondary antibodies at the dilutions indicated in the Supplementary Material (Table S1).

Image Studio Lite 5.2.5 software (LI-COR Biosciences, Lincoln, NE, USA) was used to quantify the optical density of the bands. The densitometry results were expressed as semiquantitative optical density (in arbitrary units) of blot bands. Variations in the levels of the typical housekeeping proteins (GAPDH, β-actin, and α-tubulin) were found, so Ponceau S staining was used to ensure equal loading [34,35,36].

#### 2.2.4. Proteasome Activity

Proteasome activity was assessed using a Fluorometric Proteasome Activity Assay Kit from Abcam (Cambridge, UK) based on the detection of the fluorophore 7-amino-4-methylcoumarin (AMC) after its cleavage by the chymotrypsin-like activity of the proteasome, following the manufacturer’s instructions.

#### 2.2.5. ATP Measurement

The Adenosine 5′-Triphosphate Bioluminescent Assay Kit from FLAA, Sigma-Aldrich (Saint Louis, MO, USA) was used to determine intracellular ATP levels, following the manufacturer’s instructions. The assay measured light emission with a SIRIUS luminometer based on ATP consumption that occurs when luciferase catalyzes the oxidation of D-luciferin. ATP concentrations were expressed as nmol ATP/mg protein.

#### 2.2.6. Apoptosis

Caspase-3/7 activity was analyzed in PBMC lysates using the Caspase-Glo^®^ 3/7 Assay from Promega (Madison, WI, USA) according to the manufacturer’s recommendations and as previously described in detail [24].

#### 2.2.7. Statistical Analysis

The data obtained were analyzed using GraphPad Prism 6.0 software (San Diego, CA, USA). To verify that the distribution of men and women by experimental group did not show significant differences, a chi-square test was performed. For the rest of the statistical tests, we first checked the normality of the experimental groups using the Kolmogorov-Smirnov test. If the data followed a normal distribution, a one-factor analysis of variance (ANOVA) to test for possible differences between experimental groups was used, and, subsequently, the Tukey posttest was applied to analyze detailed differences between groups accepting a significance level for *p* ≤ 0.05. In the cases of data with a non-Gaussian distribution, the Kruskal-Wallis test was used, and, subsequently, Dunn’s multiple comparisons test was applied to analyze differences between groups, accepting a significance level of *p* ≤ 0.05. Representations of experimental results are presented as the mean ± standard error of the mean (SEM).

## 3. Results

### 3.1. Widespread Oxidative Stress

**Figure 1:**
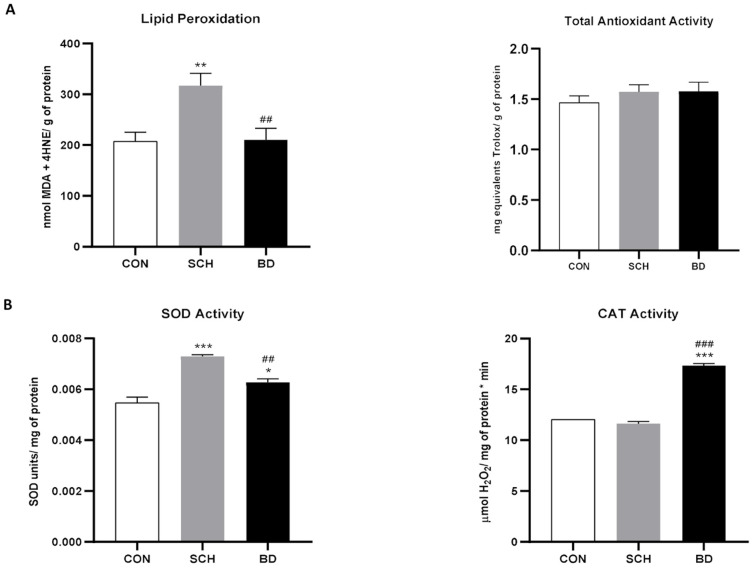
Oxidative stress status of control individuals (CON) and patients with schizophrenia (SCH) and bipolar disorder (BD). (**A**) Lipid peroxidation (LPO) (expressed as nmol MDA + 4-HNE/g protein) and total antioxidant activity (TAA) (expressed as mg equivalents Trolox/mL). (**B**) Superoxide dismutase (SOD) activity (expressed as SOD units/mg of protein) and catalase (CAT) activity (expressed as µmol H_2_O_2_/min mg of protein). Data are represented as the mean ± SEM. * CON vs. SCH or BD; # SCH vs. BP. Number of symbols marks the level of significance: 1 for *p* < 0.05, 2 for *p* < 0.01, and 3 for *p* < 0.001.

As no significant sex differences were found between the distribution of men and women per experimental group, the rest of the statistical analyses were performed jointly. To investigate the systemic oxidative profile, we analyzed TAA, SOD, CAT, and LPO. TAA showed a clear upward trend from controls to BD, with SCH patients presenting intermediate values (Figure 1A). However, LPO was significantly higher in SCH (*p* < 0.01) than in the other two groups, which showed no differences between them (Figure 1A). These data seem to indicate a higher level of oxidative stress in patients with SCH in comparison with BD patients who also have a higher antioxidant defense. However, the main line of defense against oxidative stress is constituted by antioxidant enzymes [37], mainly SOD and CAT, whose efficacy is developed in tandem. The results obtained explain the LPO in SCH, since these patients also exhibited a significant increase in SOD (*p* < 0.001) (Figure 1B), together with unaltered levels of CAT compared to controls (Figure 1B). Moreover, BD led to increased activities of both antioxidant enzymes (SOD *p* < 0.05; CAT *p* < 0.001) (Figure 1B), which favors the adequate protection against free radicals observed at the level of LPO.

### 3.2. Resulting Inflammation

**Figure 2:**
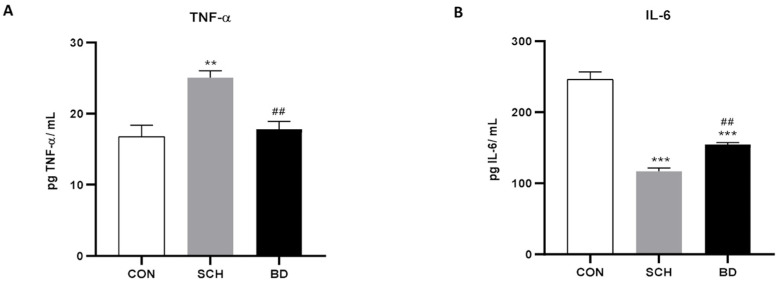
Inflammatory response in plasma of CON and patients with SCH and BD. (**A**) IL-6 and (**B**) TNF-α levels (expressed as pg of protein/mL). Data are represented as the mean ± SEM. * CON vs. SCH or BD; # SCH vs. BP. Number of symbols marks the level of significance: 2 for *p* < 0.01, and 3 for *p* < 0.001.

The main cytokines of the inflammatory cascade were studied, showing a significant increase in TNF-α levels in schizophrenic patients (*p* < 0.01), while those with BD showed intermediate levels between SCH and the control group (Figure 2A). However, IL-6 showed the opposite profile, with minimal levels in SCH (*p* < 0.001) being increased in patients with BD (*p* < 0.001) but still maintaining significant differences from the controls (Figure 2B).

### 3.3. Mitochondrial Bioenergetics 

**Figure 3:**
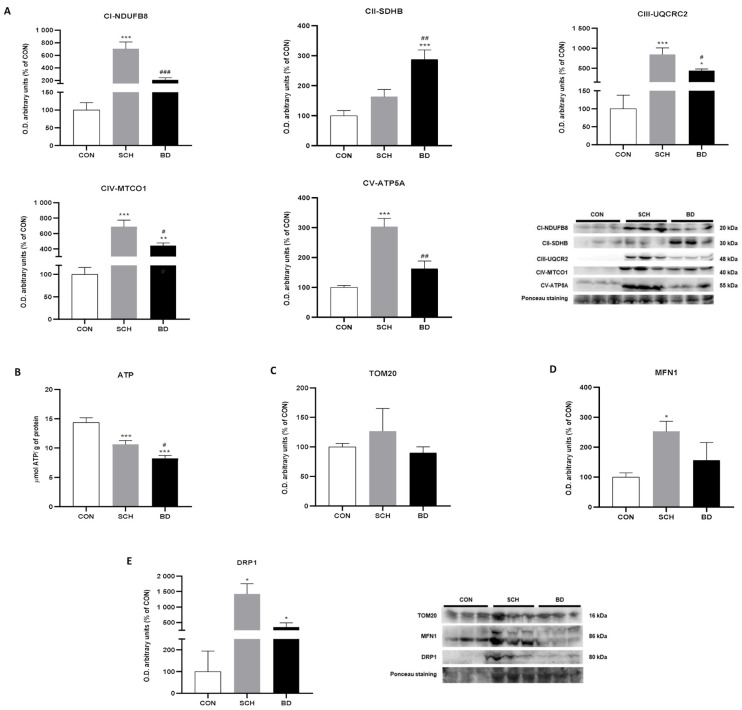
Oxidative phosphorylation profile and mitochondrial dynamics in peripheral blood mononuclear cells (PBMC) of CON and patients with SCH and BD. (**A**) Levels of subunits from the protein complexes of the mitochondrial electron transport chain (NADH dehydrogenase (ubiquinone) 1b subcomplex 8 (NDUFB8) from complex I; iron–sulfur subunit (SDHB) from complex II; ubiquinol cytochrome c reductase core protein II (UQCRC2) from complex III; cytochrome c oxidase subunit I (MTCO1) from complex IV, and ATP synthase subunit α (ATP5A) from complex V) (expressed as optical densities (O.D.) arbitrary units). Representative images of western blots. Ponceau staining was used as loading control. (**B**) ATP content (expressed as µM ATP). (**C**) Quantification of TOM20 expression (expressed as O.D. arbitrary units). (**D**) Protein levels of MNF1 (expressed as O.D. arbitrary units). (**E**) Levels of DRP1 (expressed as O.D. arbitrary units). Representative images of western blots. Ponceau staining was used as loading control. Data are represented as the mean ± SEM. * CON vs. SCH or BD; # SCH vs. BP. Number of symbols marks the level of significance: 1 for *p* < 0.05, 2 for *p* < 0.01, and 3 for *p* < 0.001.

Given that mitochondrial dysfunction promotes oxidative stress and is considered an early event in neurodegeneration [38], we next evaluated the main subunits of the mitochondrial electron transport chain that comprises mitochondrial complexes I to V (CI to CV). CI, CIII, CIV, and CV showed the greatest increase in SCH, with highly significant differences in relation to the controls (*p* < 0.001 for CI, CIII, CIV, CV) and with more damped but significant differences compared to the BD group, which presented intermediate values (*p* < 0.001 for CI; *p* < 0.05 for CIII and CIV; *p* < 0.01 for CV) (Figure 3A). These striking differences overshadowed the increases observed in BD, which, however, were almost always significant compared to controls (*p* < 0.001 for CII; *p* < 0.05 for CIII; *p* < 0.01 for CIV).

Curiously, CII showed a clearly different pattern, in which the BD group reached a maximum value compared to SCH and especially to controls (*p* < 0.01 versus SCH; *p* < 0.001 versus CON). Individuals with SCH did not show significant differences compared to the controls (Figure 3A).

ATP production, as expected, showed higher levels in controls, with significant differences in comparison to SCH (*p* < 0.001), which presented intermediate levels, which, in turn, were significantly higher than those observed in BD (*p* < 0.001 versus CON; *p* < 0.05 versus SCH) (Figure 3B). TOM20, a translocase of the outer membrane and mitochondrial marker par excellence, was found to be increased in SCH, although without significant differences from the rest of the study groups (Figure 3C).

### 3.4. Mitochondrial Dynamics

Mitochondria are highly plastic organelles that undergo fusion and fission processes to maximize their bioenergetic capabilities in stressful situations. Mitofusin 1 (MNF1), the main marker of mitochondrial fusion processes, showed the highest protein expression in SCH, exhibiting significant differences from the controls (*p* < 0.05). However, BD presented intermediate levels between controls and SCH without significant differences from either of them (Figure 3D). Moreover, dynamin-related protein (DRP1), a marker of mitochondrial fission processes, displayed an analogous graph, with a clear significant increase in SCH compared to the other two groups (*p* < 0.05) (Figure 3D).

### 3.5. Endoplasmic Reticulum Stress

**Figure 4:**
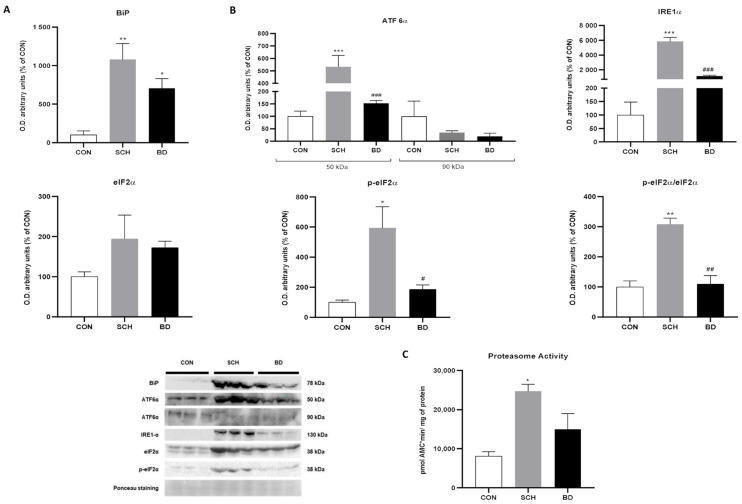
Unfolded protein response (UPR) signaling pathways in PBMCs of CON and patients with SCH and BD. (**A**) Protein levels of BiP (expressed as O.D. arbitrary units). (**B**) Quantification of ATF6α, IRE1α, phospho-eIF2α, and eIF2α expression and p-eIF2α/eIF2α ratio (expressed as O.D. arbitrary units). (**C**) Proteasome activity ratio (expressed as pmol AMCx min/mg of protein). Representative images of western blots. Ponceau staining was used as loading control. Data are represented as the mean ± SEM. * CON vs. SCH or BD; # SCH vs. BP. Number of symbols marks the level of significance: 1 for *p* < 0.05, 2 for *p* < 0.01, and 3 for *p* < 0.001.

Since oxidative stress, as well as alterations in energy production, have been linked to ER stress and the UPR, additional analyses were conducted to evaluate these pathways. First, BiP/GRP78, a chaperone involved in controlling the quality of the ER, was studied and showed significantly increased expression in SCH (*p* < 0.01) and BD (*p* < 0.05), which was more pronounced in the former (Figure 4A). 

### 3.6. Unfolded Protein Response

In the UPR study, the IRE1α pathway was found to be significantly activated in SCH compared to the other two study groups (*p* < 0.001). BD showed only a slight increase with no significant difference from the control group (Figure 4B). In addition, the ATF6α pathway, which is activated by cleavage of the peptide that transforms the native 90-kDa protein into a 50-kDa active protein, showed a much higher concentration of the inactive peptide in the controls. In fact, the active protein levels in SCH reached significant differences compared to in controls and BD (*p* < 0.001); the latter did not reach significant differences, although its value increased slightly (Figure 4B). Likewise, the activated PERK pathway represented by the phosphorylation of eiF2α and the p-eIF2α/eiF2α ratio showed, unanimously, high levels in SCH (*p* < 0.05 and *p* < 0.01), while BD presented intermediate levels (Figure 4B).

### 3.7. Proteasomal Capacity

The proteasome is in tight collaboration with the ER in the physiological degradation of proteins that the ER has not been able to synthesize successfully. A properly functioning proteasome could neutralize ER stress at an early stage. Its activity seemed to be increased in both disorders, although it only became significant in SCH (*p* < 0.05) (Figure 4C).

### 3.8. Autophagy Compensation

**Figure 5:**
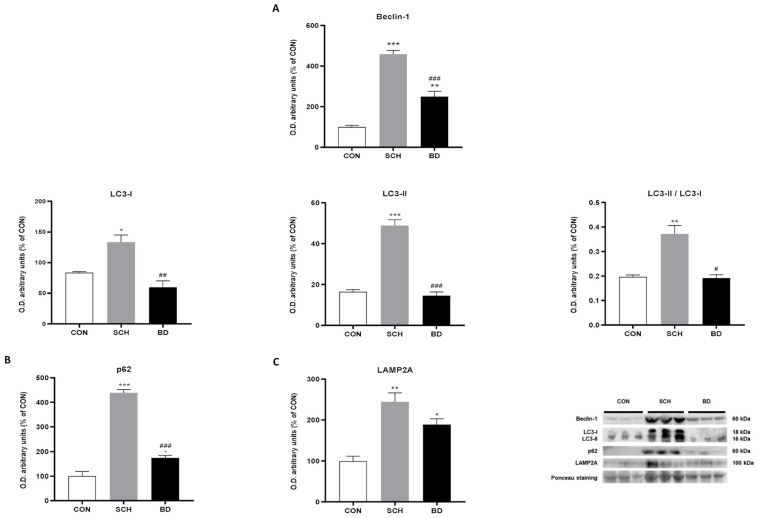
Autophagic response in PBMCs of CON and patients with SCH and BD. (**A**) Protein levels of Beclin-1, LC3-I, and LC3-II proteins and the LC3-II/LC3-I ratio (expressed as O.D. arbitrary units). (**B**) p62 protein expression (expressed as O.D. arbitrary units). (**C**) LAMP2A protein expression (expressed as O.D. arbitrary units). Representative images of western blots. Ponceau staining was used as loading control. Data are represented as the mean ± SEM. * CON vs. SCH or BD; # SCH vs. BP. The number of symbols marks the level of significance: 1 for *p* < 0.05, 2 for *p* < 0.01, and 3 for *p* < 0.001.

The main macroautophagy markers Beclin-1, LC3-I, and LC3-II (Figure 5A), their respective ratio LC3-II/LC3-I (Figure 5A) and even the autophagosome accumulation marker p62 (Figure 5B) showed analogous plots, with clearly significant maximum levels in the SCH (*p* < 0.001 for Beclin-1, LC3-II and p62; *p* < 0.05 for LC3-I; *p* < 0.01 for LC-3-II/LC3-I), while the one corresponding to BD showed lower levels and, in most cases, were similar to those observed in the control group. In the case of chaperone-mediated autophagy, the highest levels also corresponded to SCH (*p* < 0.01), but, in this case, the BD group did not present significant differences compared to SCH, and both levels were much higher than those obtained for the control group (Figure 5C).

### 3.9. Apoptosis through Autophagic Failure

**Figure 6:**
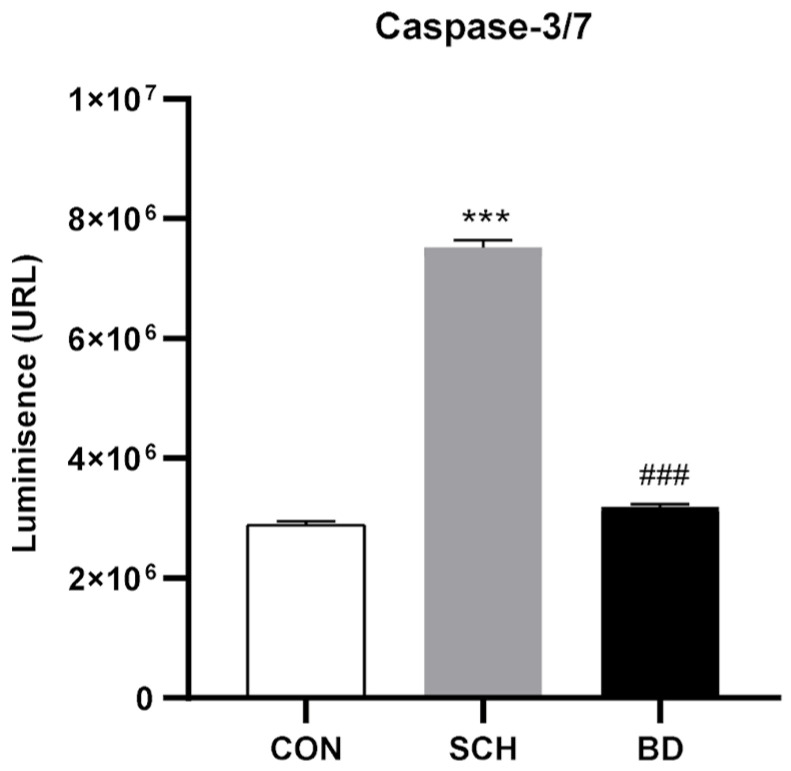
Caspase-3/7 activity in PBMCs of CON and patients with SCH and BD (expressed as luminescence (URL)). Data are represented as the mean ± SEM. * CON vs. SCH or BD; # SCH vs. BP. Number of symbols marks the level of significance: 3 for *p* < 0.001.

Caspases 3 and 7 are the main effectors of apoptosis triggering in the cell. The obtained relative activity data of both 3 and 7 showed a highly significant increase in their activity in the SCH group (*p* < 0.001), while the BD group showed no difference with respect to the control group (Figure 6).

## 4. Discussion

Precision medicine and the development of biomarkers have become essential objectives in mental health research because of the unparalleled support that their discovery would provide for routine diagnosis in mental health services. If, in addition, these biomarkers are sought in the cellular alterations that can occur differentially in each disorder, the discovery of these molecules can simultaneously lead to knowledge of the pathological basis of the disorder. To this end, it is appropriate to look for key molecules of mechanistic processes relevant to cell function and survival that will thus fulfill both requirements: to report alterations in mechanisms and to be potential biomarkers [39,40]. 

In the case of SCH and BD, knowledge of the molecular alterations defining each of the disorders would not only provide essential information about each of them, but also differentiating elements that would significantly improve the ability to discriminate between them, especially in their early stages, when diagnostic stability in both disorders is significantly reduced [41,42]. The important clinical and prognostic implications of high rates of misdiagnosis and, consequently, the introduction of non-specific treatments call for researchers to determine valid and reliable objective markers potentially useful in the differentiation of these disorders.

Our results have allowed us to identify essential parts of the disorder-specific cellular interactome, revealing sharp differences in their cellular development. Thus, SCH showed, in general lines, active and manifest damage superior to that detected in BD for each cellular mechanism studied. In very general terms, the addictive behaviors [43], which usually accompany the idiosyncrasy of SCH and which have been profusely described as pro-oxidants [44], could be implicated in these differences found between the two disorders, as an alternation of phases is observed in BD that could reduce the impact of the psychotic phase, providing a respite in the cellular alteration that temporarily ends up becoming significant. This confers a fundamental role to oxidative stress for its chronicity in the development and evolution of the cellular changes detected. Thus, although oxidative stress is a commonly described element in mental disorders [17], the SCH showed elevated levels of lipoperoxidative damage which, although previously described by other authors [45,46,47], were strongly supported in this work by the imbalance observed between the main enzymes SOD and CAT. An elevation of SOD that is not accompanied by a proportional increase in CAT causes SOD to act as a prooxidant by facilitating the generation of hydroxyl anions that have not been prevented by CAT and that will trigger oxidative damage to macromolecules, as has been abundantly demonstrated in the literature [48,49,50]. However, in the case of BD, the increase in CAT, the downstream enzyme, and not SOD, causes the opposite effect: increased protection against possible increases in free radicals, which may have repercussions on multiple cellular cascades and mechanisms [17,51]. The alteration of oxidative levels is closely related to subsequent inflammation, which, in the case of the two disorders studied, once again showed different results, with elevated TNF-α levels in SCH being absent in BD. Previously, other authors described a similar pattern of inflammatory action in SCH, with elevated levels of pro-inflammatory cytokines, such as TNF-α [51], together with a clear decrease in anti-inflammatory cytokines, such as IL-6 [52,53]. Again, several authors have previously related these divergences in the inflammation observed in both disorders to their differential compulsion to abuse or disordered eating, leading to an increase—sometimes exacerbated by the widely established BMI—in this disorder [52,54], which, however, is not triggered with the intensity similar to BD in our case.

The mitochondrion, the cell’s main energy producer, has in its electron transport chain the fundamental pathway for ATP synthesis and for the induction of oxidative stress, so that, in view of the previous results, the alteration of this organelle was to be expected. In fact, the components of this chain have shown very different results in both disorders. Thus, patients with SCH disorder presented a significant increase in the expression of most of the components of the chain, and this increase was easily related to an inefficient energy synthesis or an increased energy requirement, facts that have been previously described in obesity [55] or in other mental disorders [56]. However, attempts to improve their energetic capacity were unsuccessful in view of their ATP production, which was much lower than in control individuals. BD, on the other hand, showed a striking increase only in complex II of the transport chain, the only one reduced in SCH. This specific increase in complex II has been observed previously in multiple pathologies [21,24] and is associated with damage to complex I, which can be replaced by complex II, maintaining the capacity of the electron transport chain to act, although at the cost of a drastic reduction in energy efficiency (1 ATP produced through complex II for 10 ATP through complex I). This would explain the minimal ATP levels detected in BD patients. Data on mitochondrial dynamics, essential to complete mitochondrial pathophysiology because of its role in maintaining the function of this organelle [57,58], continue to maintain the pattern of greater involvement in SCH than in BD, revealing an overexpression of proteins involved in both fusion processes, commonly related to optimization of mitochondrial function [59] and fission triggered by clear massive mitochondrial damage [60]. These mitochondrial fusions, rather than mitochondrial fissions, are probably behind the poor success in increasing mitochondrial number visualized by TOM20 in SCH following increased expression of complexes involved in oxidative phosphorylation.

Adequate ATP production has been shown to be crucial for protein homeostasis, and the data obtained lead us to suspect an impairment, albeit differential, of cellular protein synthesis in these disorders. Although recent studies have identified mitochondrial dysfunction in patient samples and in animal models of various neuropsychiatric disorders, little is known about this step following energy disruption. The BiP/GRP78 protein plays a key role in signal translation through the formation of different protein complexes in the ER lumen. Its highly significant increase in SCH seems to indicate important non-native conformational alterations in proteins, as already described in other pathologies [61], which must be degraded by the proteasome as a first clearance mechanism [62]. The high degree of proteasome activity detected in SCH supports this hypothesis. If proteasome action is insufficient, BiP/GRP78 is also a key regulator of the ER stress response through activation of the UPR [63]. The unanimously elevated response of the three UPR pathways in SCH patients compared to the much lower level in BD patients is probably the most important indicator of the aberrant situation in the cellular interactome of SCH patients in our study, as well as the most important source of biomarkers. Activation of the three UPR pathways leads to the triggering of a large number of signaling cascades ranging from chaperone production to blocking protein synthesis in an attempt to restore cellular homeostasis [64]. Likewise, overactivation of the UPR over time has been associated with several neurodegenerative diseases, and although its inhibition has been put forward as a possible therapeutic measure for the treatment of these diseases [65], its central role as a regulator of the cell in a crisis situation seems to argue against it.

The imbalance between UPR-triggered cellular cytoprotection mechanisms and the increase in misfolded proteins leads to the triggering of autophagy as a mechanism for cellular clearance and destruction of protein aggregates; they are generated from aberrant proteins, are blockers of cell communication, and present in a large number of pathologies [66,67]. When autophagy is insufficient to restore cellular balance, autophagosomes accumulate and, with it, their waste products, so the autophagosomal marker of damaged proteins, p62, is an excellent indicator of autophagic blockage [25]. This is the case observed in SCH patients who, although they showed increased autophagosomal activity, were not able to reduce the significantly elevated levels of p62 compared to BD patients who appear to be oblivious to the exponentially developed cellular damage in SCH. This autophagic overactivation, which gradually blocks the mechanism, has been previously observed in other pathologies in our laboratory [21] and others [68], ultimately leading to cell death by apoptosis, which was also found in SCH patients. This induction of apoptosis by autophagic reduction or blockade has been described in other neurological disorders [69] and even in SCH models [70], although this is the first time it has been described in patients.

Mitochondrial damage [71], altered protein synthesis, the response to this alteration [72], and even autophagy blockade and finally apoptosis [73] have been extensively linked in each case to increases in free radicals. In this article, we have found that, although being the cause or the consequence, increased oxidative stress is a constant at the cellular level in both disorders, showing a significantly higher incidence in BD compared to SCH and acting as a real link between these alterations.

These results have maintained their significant differences between the two disorders even when the medication prescribed for each of them was far from similar, so the changes observed at the cellular level in the key molecules studied appear to be strongly disorder-dependent and/or poorly modulated by treatment.

Although this is a preliminary study, which implies a small sample size limited to a specific geographic area, the results obtained are so relevant in terms of statistical significance and, therefore, of such relevance for clinical practice, that, considering their possible global projection, it would be of interest to reproduce studies that include a larger number of individuals from different areas, ethnicities, and cultures that would favor the potential validation of the universality of these biomarkers.

## 5. Conclusions

The results obtained from very heterogeneous groups of patients with different ages and sexes not only allow us to show the way to identify potential disease-specific biomarkers to target future clinical trials in large and varied populations, but also demonstrate how both pathologies use different cellular strategies of response to alterations, which deepens the knowledge of the differential cellular mechanisms involved in each disorder. Thus, the SCH patient, perhaps affected by increased oxidative stress, shows marked cell damage that favors the triggering of homeostatic tools for cell recovery and survival, which is clearly insufficient since cell death is triggered in a highly significant way. The BD patient, on the other hand, perhaps due to the cyclicity of its phases, has less oxidative damage and inflammation [74], decreases general markers, and shows a much milder induced damage, with no practical activation of survival mechanisms and, therefore, inhibition of apoptosis. Alterations in cellular mechanics are the basis for the development of any pathology, and diseases based on damage at the cellular level, e.g., mitochondrial [75] and lysosomal [76], are clearly devastating for the individual suffering from them. These alterations, which could be caused by or be a consequence of oxidative stress, create biological fingerprints and protein changes, which can subsequently be tracked early with diagnostic, prognostic, or therapeutic interest through broad, varied, and even clinical assays. Therefore, we believe that this study lays the necessary foundation for detecting potent biomarkers of both disorders. Therefore, the development of molecular psychiatry, the normalization of symptoms, and the transformation of disorders into pathology will allow not only a better approach and treatment of these serious mental disorders, but also their normalization at a social level and the reduction of the stigma attached to them.

## Figures and Tables

**Table 1:** Demographic and clinical characteristics of control individuals (CON) and patients diagnosed with schizophrenia (SCH) and bipolar disorder (BD).

	Controls Mean ± SEM or %	Schizophrenia Mean ± SEM or %	Bipolar Disorder Mean ± SEM or %
Men; women	35.7%; 64.3%	62.5%; 37.5%	50%; 50%
Age (years)	45.7 ± 4.1	46.7 ± 2.7	50.0 ± 4.0
BMI (kg/m^2^)	-	29.5 ± 1.3	29.43 ± 1.5
Years of diagnosis	-	19.8 ± 2.9	14.5 ± 2.7
Age at diagnostic	-	26.0 ± 1.3	38.15 ± 4.6
Disability			
Yes	-	76.2%	46.1%
No	100%	23.8%	53.8%
Antipsychotic			
Aripiprazole oral	-	5.9%	13.3%
Aripiprazole IM	-	2.9%	-
Clozapine	-	14.7%	-
Levomepromazine *	-	2.9%	-
Olanzapine	-	23.5%	-
Paliperidone oral	-	11.8%	6.7%
Paliperindone IM	-	23.5%	26.7%
Quetiapine #	-	5.9%	33.3%
Risperidone	-	8.8%	20.0%

BMI: body mass index; IM: intramuscular. * Only 1 patient is taking typical antipsychotics. # Quetiapine is prescribed primarily as a hypnotic. All patients, except one (300 mg), have been prescribed doses ranging between 25 and 100 mg.

## Data Availability

No new data were created or analyzed in this study. Data sharing is not applicable to this article.

## Supplementary Materials

The following supporting information can be downloaded at: https://www.mdpi.com/article/10.3390/antiox12111948/s1, Table S1: antibodies.
